# Serious Games as a Validation Tool for PREDIS: A Decision Support System for Disaster Management

**DOI:** 10.3390/ijerph192416584

**Published:** 2022-12-09

**Authors:** Sara Rye, Emel Aktas

**Affiliations:** 1 School of Business, Department of Innovation, Leadership, Strategy and Management, Southwark Campus, London South Bank University, London SE1 0AA, UK; 2 Department of Logistics, Procurement and Supply Chain Management, Cranfield University, Bedford MK43 0AL, UK

**Keywords:** decision-making, disaster response, DSS, simulation game, proliferation of suppliers, serious game

## Abstract

In this paper, we validate PREDIS, a decision support system for disaster management using serious games to collect experts’ judgments on its performance. PREDIS is a model for DISaster response supplier selection (PREDIS). It has a PREDictive component (PRED) for predicting the disaster human impact and an estimation component to Estimate the DISaster (EDIS) needs to optimise supplier-based resource allocation. A quasi-experiment design embedded in a participatory simulation game is conducted to compare the opinions of equal samples of 22 experts and non-experts. The following questions are put forward. First, “Does PREDIS model assists the decision makers to make the same decisions faster?” Second, “Does the PREDIS model assist the non-experts as simulated decision makers to decide like an expert?” Using AHP weights of decision makers’ preferences as well as Borda counts, the decisions are compared. The result shows that PREDIS helps to reduce the decision-making time by experts and non-experts to 6 h after the disaster strike, instead of the usual 72 h. It also assists 71% of the non-experts to make decisions similar to those made by experts. In summary, the PREDIS model has two major capabilities. It enables the experts and non-experts to predict the disaster results immediately using widely available data. It also enables the non-experts to decide almost the same as the experts; either in predicting the human impact of a disaster and estimating the needs or in selecting suitable suppliers.

## 1. Introduction

The first official report about the disaster human impact including fatality, injured and homeless population in disaster area, is released within 72 h to three weeks after a disaster strikes. This report released by the UN is called the Multi-Cluster/Sector Initial Rapid Assessment (MIRA) report. However, the decision about resource allocation and life-saving activities needs to be taken before the MIRA report [1]. In the absence of the real time data, a model called PREdictive model for DISaster response supplier selection (PREDIS) was introduced by authors previously [2,3,4,5,6]. This paper aims to validate this model using an experimental technique called the simulation game. 

PREDIS predicts the human impact and estimates the resources required. It also assists in selecting the humanitarian-response suppliers. This Decision Support System (DSS) is a combination of a PREDictive component (PRED) for predicting the disaster human impact [6] and an estimation component to Estimate the DISaster (EDIS) needs [5] to optimise supplier-based resource allocation. 

We validate PREDIS through a serious game simulation to compare the result of decisions made through PREDIS by experts and non-experts [7]. Validation increases the confidence of using a model [8] through practice, tests, and evaluations which leads to a reduction in cost and time [9]. For a DSS, the validation can be obtained through repeated testing by unbiased agents [10]. The consistency of the result of DSS in the past has been established based on a set of interviews from the unbiased agents through methods such as Analytical Hierarchy Process (AHP) [11] or through Agent Based Simulation (ABS). These methods examine and formulate the behaviour of the real world decision-making entities [12,13]. 

We examine the validity of PREDIS through a simulation game to compare the empirical model to the real performance of the system (here disaster response). Simulation is defined as a representation of a real-world environment, to imitate the system or process overtime, where the direct scientific observation of the real system due to inaccessibility, cost or danger is impossible [14,15]. This is the case for PREDIS, as it is impossible and unethical to create a disaster few times and observe how the decision makers allocate resources or manipulate the affected population to examine the validity of the model. 

To that end, a participatory simulation [7,16] also called a simulation game [17] or a serious game [18,19] is used in this paper by asking the participants to take decisions based on underlying rules that are consistent with the real world disaster scenario [8]. The research questions are twofold. First, “Does PREDIS model assists the decision makers to make the same decisions faster?” Second, “Does the PREDIS model assist the non-experts to decide like an expert?” To answer the above questions the paper is outlined as follows. First a review of literature presents the application of simulation games in validation of DSSs in general and disaster management in particular. The data section outlines input and output data, the source, and combinations followed by the method section where the process of data analysis is highlighted. The results summarise the findings followed by conclusion and limitation of the research. 

## 2. Literature Review

Effective disaster management relies on the accuracy of data as well as communication with end-users and optimised resource allocation decision [20]. The optimised decisions can be simulated within a DSS [21,22,23]. The simulation games are widely used in operations management [24,25]. They range from simple red bead experiments [26] to system simulations like the Beer game [27,28] and Cuppa Manufacturing games [29] to complex interactive environments. A myriad of simulation games are introduced for humanitarian logistics and disaster situations, for training [30], crisis management [31] and assessing natural risk management [18,20].

FloodSim (Playgen.com, 2014, accessed on 16 February 2018) is a simulated game where the player is in charge of all flood-related policy-making decisions for the next three years in the UK. FoodForce (foodforce2.com, 2014, accessed on 16 February 2018) is another game in which players take on missions to distribute food in a famine-affected country. In ‘Stopthedisaster game’, the players make decisions leading to the reduction of disaster risk (Stopdisastersgame.org, 2014, accessed on 16 February 2018). In Darfurisdying (Darfurisdying.com, 2014, accessed on 16 February 2018), players try to survive in a refugee camp. Planning with Large Agent-Networks Against Catastrophes (Plan-C) software is a simulation program with the ability to cover 1,000,000 injured. It provides statistical outcome data at medical, emergency responder, and community levels. This model is tested on food poisoning and terrorist attack modelling [32]. 

These games are useful for planning and familiarising decision makers with the decision-making process in a disaster situation. They also either heavily rely on resources such as computers as a medium for simulation or require the design and production of non-computerized games (such as board game or card games) as well as training for facilitators to be able to effectively moderate and practice the games.

Simulation games are used for validating the DSS in variety of subjects. These are mainly designed to observe the behaviour of the players and as a result assesses the effectiveness of the model. For example, a model about the medical treatment [33] investigates how the patients’ knowledge would change their decision about treatment. An agent-based model of an entrepreneurial game [22] can develop a comprehensive entrepreneurial mind in the user. 

Simulation games have also been used for cross-cultural DSS [34] about distribution, supply chain, and operations. Some have been used to validate a model on city logistics [8] to collect information about the behaviour and beliefs of the decision makers on the profit margin and supply suppliers. A simulation game is used to validate a model in land use [35] to analyse the decisions made by the households with a quasi-experiment to see if they would change their decision about land for environmental/financial rewarding behaviour. 

The validity of these models in a variety of cases are tested through questioning and comparing the results from the experts and non-experts. The examples include models on mapping and hydraulic testing data on construction areas [36], information technology [37], development and testing of linkages between supply chain relationship in performance [38], behaviour of the brain fluid to validate a brain model [39], even social sciences [40], or testing immersive design tools [41] through questioning the participants. 

Simulation games are so successful that in some large oil and gas projects the project management team use integrated dynamic simulation-based solutions throughout the project lifecycle not only to validate the design but also for operator training and start-up support amongst other uses [42]. In this context, the main goal of the game is to simulate the actors’ decision-making processes. This leads to the demonstration of the consequences within social systems where the users must cope with difficulties arising from the complex nature of these systems [43]. 

Comparing expert and non-expert decisions [44] for the purpose of validation through a simulation game has precedents within the scientific and technological forecasting, medical and managerial decision-making, quality assessment and operational research, or validation of a cognitive capability model through expert opinion [45] or validation of safety behaviour [46] or practitioners’ behaviour in the field of human resources [47].

To validate the PREDIS model, the latter approach is adopted. In present research a non-computer-based simulation game is designed for implementing the decision-making model for supplier selection in disaster situations, in a simulated process within two groups of the experts and non-experts to answer the research questions. The process is outlined as follows.

## 3. Data

**Figure 1:**
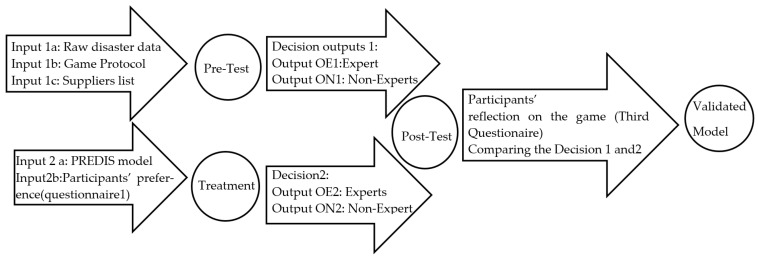
Input/Output Data.

Various sets of primary and secondary data are collected in the paper as outlined below. The secondary data utilised here includes a panel data gathered through the PREDIS framework as well as the result (including prediction of the affected population as well as the estimation of the resources required, coupled with the optimisation of the resource allocation) produced through PREDIS model. The primary data includes the result of the two questionnaires conducted within this study as well as the result of the simulation game. The first questionnaire produces the preferences of the decision maker. The second questionnaire collects the opinion of the participants (separately for experts and non-experts) about the simulation game as well as PREDIS. The input/output are demonstrated in Figure 1 including the following data sets.

**Figure 2:**
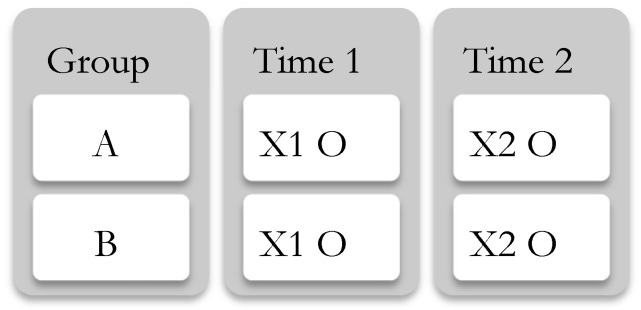
Schematic of the counterbalanced design adopted by authors.

**Figure 3:**
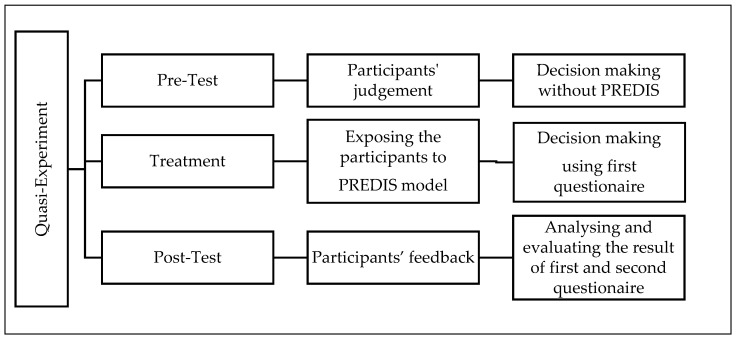
The quasi-experiment design of the study. Adapted by authors from [72,77].

**Figure 4:**
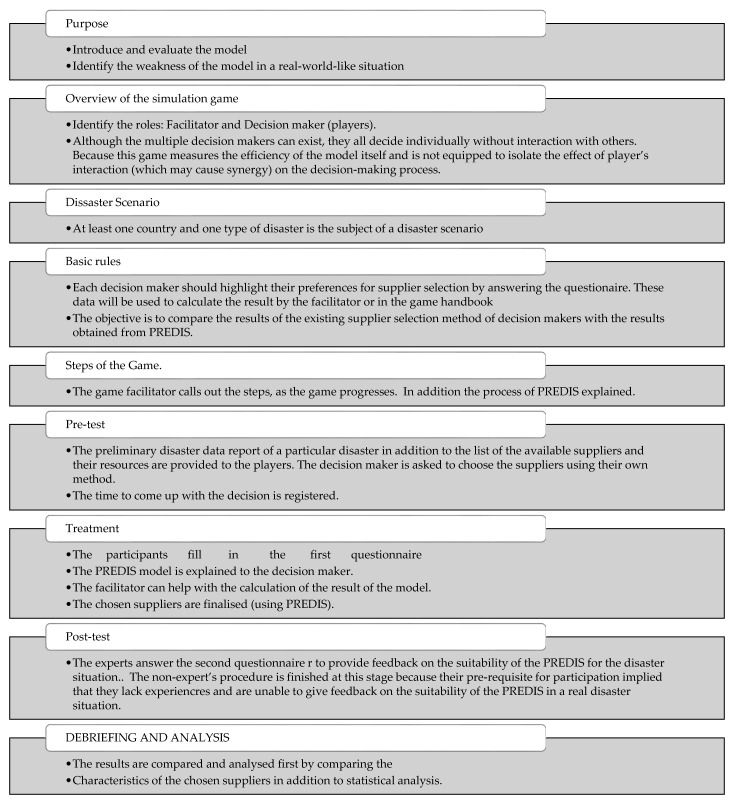
The game Protocol.

Figure 1 shows the data collection in three phases of the study (Pre-test, treatment, and post-test) depicted in the design on Figures 2 and 3. Input (1a) includes disaster raw data such as Panel data, secondary data collected through PRED illustrated in Table 1]. Also Input (1b) in Figure 4 includes the game protocol, and Input (1c) includes the list of humanitarian suppliers. This suppliers who can provide the resources required are adapted from EDIS in Table 2, and based on the steps in Table 3. This therefore provide the participants’ preferences including Questionnaire in Table 4, first set of primary data collection. The process of the data analysis through pre-test which will be described in methodology, leads to a set of Decision Outputs (1) including decisions from Expert (OE1) and Non-Experts (ON1). Another set of data is utilised in Input (2a) which includes first questionnaire in Table 5 [primary data collected through a quasi-experiment]. This leads to a set of decisions outputs (2) including decisions from Expert (OE2) and Non-Experts (ON2) in Table 7 [result processed through PREDIS in Table 8], Tables 9–11. A final round of questionnaire then collects the primary data as an input along with the OE1,2 and ON1,2 to compare and analyse the result of the decisions made above Tables 12 and 13, leading to the validation of the model as the final output. 

### 3.1. Secondary Data

Pre-test utilises the raw secondary disaster data which classifies disaster scenarios compiled together to provide a set of panel data at the country level drawn from PRED [6]. PRED uses the prominent natural disasters occurring after 1980 mentioned in the Encyclopaedia of Disasters [48] including 32 disasters. The result was compared to the 10 costliest and 10 deadliest disasters in NatCatSERVICE (Munich RE, 2007) leading to a more complete list of disasters. The data were next compared to the EM-DAT and Munich RE, accumulating to 4252 disasters. This process required a definition of the target population, the time period under investigation and the variables of interest [49]. Based on the EM_DAT definition, only disasters that have affected more than 10 people, and were declared in need of international assistance we considered. An example of this dataset is illustrated in Table 1.

Table 1 includes data about Disaster Number provided by EM-DAT, impact and end time, the type of the disaster, the country of incident, its population and its population density collected from EM-DAT as well as the number of killed and total affected population. The human development index (HDI) drew from and disaster risk index or DRI [50]. Pre-test also utilises the secondary data of suppliers’ list drawn from EDIS [5]. An example of this dataset is provided in Table 2. 

Table 2 shows the data adapted from EDIS which shows the anonymised list of humanitarian supply partners who possess the resources required for affected population in disaster situation. This resources are classified based on humanitarian clusters of WASH, Nutrition, Shelter, Health [51].

### 3.2. Primary Data

The primary data collection took around 3 weeks to complete through two questionnaires, extending from pre-test (questionnaire one) to post-test (questionnaire two). The ethics approval was obtained through the ethic committee of the Brunel University. The sample population include two sets of experts and non-experts. The logic behind the segregation between expert and non-expert participants is that in many cases in disaster situation, the people who are forced to decide about relief aid, in NGOs or voluntary organisations, amongst others, are non-experts. If the model can produce a comparable result of decisions between experts and non-experts, it is possible to argue that the model can help the non-experts to decide like experts.

#### 3.2.1. The Characteristics of the Participants

To address the above, two groups of participants separately participate in this simulation game. The prerequisite of group one is that the participants have at least one experience in decision-making in a disaster situation. These participants are summoned from humanitarian groups and voluntarily participated in the game. The information about the experiment and invitation for expert participation was distributed amongst various organisations (Environment agency, Crisis departments of five different embassies, Business continuity departments of Munich RE, Barclays Bank and Lloyds bank, and individuals who had connections with humanitarian organisations including UN, UNISDR, UNICEF, World Vision, Caritas International, British Red Cross, American Red Cross, Save the children and various specialised forums and groups related to disaster management on LinkedIn (including Business Continuity and Disaster Recovery Professionals, Business Continuity Management & Risk, Business Continuity/Disaster Recovery Network, Disaster & Emergency Management, Disaster, Disaster, Disaster Management—Multi Hazard Risk Assessment, Disaster Researchers and Disaster Management Professionals, Disaster Risk Management Practitioners, Emergency Preparedness Consultants/Trainers Group, GWU Institute for Crisis, Disaster and Risk Management, Humanitarian & Disaster Response Technology Network, Innovations in Disaster Management and Emergency Response!, Natural disasters and natural hazards, Natural Hazards and Disaster Risk Management, Performance Management, Professionals in Emergency Management, World Conference on Disaster Management) in addition to humanitarian summit, Risk analysis conference, OR society conference and UCL IRDR society.

Twenty-two experts participated in the research. These experts were from various backgrounds in different governments, international humanitarian organisations, NGOs, disaster consultancy professionals and corporate continuity departments in addition to military officers and fire brigade members. The prerequisite of group two is that the participants have no experience in disaster response and voluntarily participated in the game. To make the non-expert groups comparable to the experts, an equal number of non-experts were invited by distributing invitations to various graduate and undergraduate students (by contacting their lecturers) in various areas of studies including but not limited to management, Operational Research, disaster management, history, actuarial sciences, law and biology. In addition, the invitations were sent to any non-students who were interested in participating including engineers, HR professionals, MDs of private companies, health care managers, legal aid, high school teachers, social activists, and carpet designers. These contacts were made through the first author’s personal circle of acquaintances, and they were asked to forward this information to anyone they suspect might be interested. To keep the group comparable to the experts, the author collected data from 22 non-expert participants.

#### 3.2.2. The Process of Data Collection

The sessions were held in one to one virtual appointments, which took place online. The duration of each session was around an hour, 15 min of which was spent watching a presentation about PREDIS framework. A booklet containing an explanation of the aims and objectives, and the consent form as well as the description of the process was sent out to the participants a week before the session. They were also asked to use their existing DSS frameworks to choose the suppliers based on their resources. Within the session, they were asked to provide their choices, none of which have made a decision. Then a power point presentation was given by the facilitator, which explained briefly, how the model works and asked if they had any questions. After the start of the session the participants were asked to provide the set of decisions they made before the session based on the questionnaire and the pack sent to them. The plan was first to discuss these decisions and any frameworks they used. However none of the participants actually came up with a list of decision (selected suppliers) or suggested any decision framework for disaster. The game protocol was then provided to the participants and the simulation game was run. The simulation game described in the next session includes two sets of questionnaires first for gathering the preferences of the two groups of participants and second for asking the opinion of the participants about the simulation game as well as PREDIS. The details are outlined in methods.

## 4. Method

Despite subjectivity of the simulation game, it might be the only viable way to examine decision making agents who try to make rational decisions [23], even though they lack the whole set of date required for a rational model [52,53]. This is due to the fact that the classical theory of rational decision making has limitation in real scenarios Simon 1972 and Jensen 2012 where all alternatives to a problem are not clear to the decision maker. In theory all the criteria for decision making must be available to evaluate and compare and finally chosen as the most preferred one. This is very unlikely in real case [54] and specifically in disaster situation where the full data set, the criteria and even alternatives are hardly known. So when the authors mention “optimise” in this paper, they mean “satisfice” through discovery and selection of satisfactory alternatives. Moreover, when the decision is subjective to the person’s preferences, this leads to an argument against simulation games, that it uses human judgment to validate the decision models designed to improve human judgment. The response to this criticism is that simulations provide a relatively flexible and realistic representation for complex problem, and major decisions are made based on the simulation results [55].

### 4.1. Grounds for Choosing the Simulation Game

Considering all the above limitations, the authors rely on two grounds for choosing the simulation game for this stage of the research. The first reason is the numerous experimental studies in the non-management areas of research, where scholars use human judgment in hypothetical situations including vignette studies and economic experiments. These two methods are elaborated further as follows.

“Vignette studies” are one of the vastly used methods, which involves presenting participants with a hypothetical scenario, and asks how participants would think, feel, and act in the depicted situation [56,57]. Vignettes are generated from a range of sources including previous research findings [58,59], in collaboration with other professionals working in the field [60]. In the field of disaster management/emergency, vignette is used for validation of real cases to accurately reflect actual practices and assess the quality of management in complex emergency situations [61] or look at the personal narrative of women where the vignettes represent individual observations to evaluate the situation of gender before and after a disaster [62]. Participants are typically asked to respond to these scenarios by answering what they would do in a particular situation or how they think a third person would respond [63].

The simulation game presented here is not a full vignette study, but combined with an experimental design for simulation game, both with merits in literature. As mentioned before the success of vignette studies in human judgment are vastly accepted as a laboratory-like tool in validating hypothetical scenarios, and thus signalling the power of similar tools. Another use of human judgment as a tool is a simulation model that replicates the decision-making process in disaster response networks. The author believes that this combination of vignette-simulation within an experimental design offers participants distance and space to provide a discursive interpretation within the context by constant interactions between the decision makers and the real-like scenarios. This has also an educational effect on the decision makers in the long run, as they will learn from their own experience by repeating the process of decision-making in a simulated environment of disaster response. Where this ‘snap-shot’ of disaster scenarios does not offer enough information for an individual to make a decision or provide an explanation, the situated context of a simulation model could work similarly to a vignette, which can be used to explore the main influencing factors.

### 4.2. Research Design

To design a simulation game for validating the PREDIS, a combination of the vignette concept into simulation game is considered as follows. Some researchers believe that simulation games are the third research methodology in line with induction and deduction [23]. To validate the PREDIS model a series of assumptions are put forward. Assumption 1 outlines “the selected suppliers through the MIRA report’s data is not significantly different from the selected suppliers through PREDIS data”. Considering the PREDIS data is available 72 h before MIRA, the acceptance of this hypothesis leads us to believe the PREDIS would make the decision making process faster and reduces delays in humanitarian aid up to 72 h. Assumption 2 is “the decisions made by the test group of experts is not significantly different from the control group of non-experts”. If these assumptions are accepted, it leads us to believe that the PREDIS model not only helps the experts to decide faster but also helps the non-experts in making decisions quality decisions like experts. So based on the above assumptions and the research questions were introduced earlier, the following propositions are explored: Proposition 1: “The PREDIS model assists the decision makers in making the same decisions faster” Proposition 2: “The PREDIS model assists the non-experts and experts equally to make similar decisions”. To test the above propositions two designs were considered. The first option was to put forward a series of questions in the frame of a vignette study. The second option was to use an experiment and practically see how the model works in the real life. These options are reviewed as follows.

Vignette design—In this technique a set of questions and scenarios are exposed to decision makers to examine the decision-making process and how they come up with the decision [64,65]. The advantages of this method are that it reduces the possibility of an unreflective response, and it is very useful when the questions are sensitive because the respondents answer the questions about the hypothetical characters and not themselves. However, this technique could not facilitate a hands-on experience for the participants where they can try the PREDIS platform. In addition, it does not provide a setting where the experts and non-experts could be compared. It also could not consider the learning effect associated with being exposed to the PREDIS model in the process of decision-making. Although the elements of a vignette study such as scenario making, survey questions and human judgment are present in this study; a pure vignette study is not appropriate. The reason is that the elements of experiments are also present in the study, where the participants are exposed to the PREDIS model.

Experiment design—The use of the experimental design in simulation games is popular due to its resemblance to the laboratory conditions [52,66,67,68,69,70,71,72]. Experimental approaches are used in various studies including laboratory experiments with hypothetical decision-making situations such as purposefully designed business simulation games, in which participants have to make entrepreneurial decisions within the systematically controlled rules of the game [68,73,74]. In an experiment design, the respondents are exposed to the PREDIS model, and their actual decision and the effect of the model are registered and compared before and after. Pre-test/post-test designs are employed in both experimental and quasi-experimental research [75]. For the purpose of this paper, a quasi-experiment design is the most suitable because not all the factors in human decision-making process could be controlled based on the principle of rational choice mentioned before. The design adapted in this part uses a non-equivalent group counterbalanced design [76] as depicted in Figure 2.

Figure 2 shows that the group A (here non-experts) and B (here experts) are a combination of non-equivalent participants distributed in two groups. The design implies that each group is exposed to the treatment ×1 (here the preliminary disaster data report), which is observed followed by being exposed to the treatment ×2 and is observed. Treatment ×1 comprises of providing the participants with a disaster scenario and asking them to choose from a list of hypothetical suppliers based on their knowledge and the data in a preliminary disaster data report. Treatment ×2 comprises of providing the participants with the PREDIS model and asking them to choose from a list of hypothetical suppliers based on the predictions in the model. For the treatment phase [77], a simulation is conducted which involves representing the situation by creating an artificial setting (here the disaster scenario case) in which individuals decisions are registered and compared. The reason being is that it is capable to create a large amount of data in a short period of time and enable access to the issues that may not be amenable to observation in real life such as problem-solving and decision-making. They also enable the researcher to create and record the situation in order to examine the effect of an intervention [77]. As illustrated in Figure 3, this design relies on obtaining a pre-test measure of the outcome of interest (here decision-making in disaster situation) prior to administering some treatment (here exposure to PREDIS model) followed by a post-test for the same measure after treatment occurs.

Following the process in Figure 3 in the pre-test phase a sample selected equally from a mixed population of experts and non-experts were asked to select the suitable humanitarian suppliers based on this historic data using whatever method and framework they prefer. The elements of experiment was introduced to the design by exposure to the PREDIS model in the treatment phase. In the post-test, the participants were asked to choose the suppliers based on PREDIS process only. The disaster scenario was presented to the participants in two stages. In the pre-test phase, the case provided in MIRA report regardless of what is required for the PREDIS model. The reason is to avoid disclosing any data about the PREDIS process to reduce the effect of pre-disposing the participants to “treatment” in the phase of “pre-test”. This also helps the decision makers to use their experience and current frameworks in a way they normally use in disaster situation without being exposed to the process of the PREDIS model. In the post-test phase the scenario is again presented to them but in the brief format required for PREDIS model. The only information required for PREDIS model is the type, date and the country of the disaster occurrence. The rest of the data is calculated by the PREDIS platform.

#### 4.2.1. Pilot Study

Before the launch of the primary data collection, a pilot study was conducted, testing the research design on one participants. This identified that the experts would not give a clear set of decisions during the pre-test. For example, when asked “please rank and choose the suppliers you would use in the given disaster situation”, in the pre-test experts would say “I would use the suppliers with whom I have had good relationships in the past”, or “I would choose the suppliers based on the quality of previous experiences” or “I will call any local supplier in the area to see if they can provide the resources”. Therefore, comparing a list of chosen suppliers in the pre-test and post-test procedure was not possible. Consequently, the author who has planned to use the Turing test [78] for comparing the development of decisions in pre/post-test. For that reason the plans changed to the comparison of the result in the post-test between experts and non-experts. Therefore, the results of the pre-test in all cases were used just to show that at the time of the research an actual framework that provides clear comparable choices is non-existent. Another change made in the design as a result of the pilot study was few changes in the questions in the post-test questionnaire. For example in the pilot study the participants’ responses to the level of experience were unrealistic. For example, a participant stated that he had 38 years of experience in disaster operations. Later it became clear that they had been providing consultancy to the humanitarian organisations on and off during the past 38 years. To differentiate between this participant and the participants who actually have been in the first line of disaster aid, three questions were asked about age, sector and the number of disasters in which they have been involved. More details about the questionnaire is found in the design section.

#### 4.2.2. Simulation Game

Simulation game design was chosen as the overarching process for the quasi-experiment. The design of the game is mapped based on the Garris [79] as presented in Table 3.

Following the above design the game protocol is put forward in Figure 4.

Following the game protocol in Figure 4 the pre-test is put forward as follows.

#### 4.2.3. Pretest

The process starts with the pre-test where the participants (An equal set of 22 experts and non-experts) individually are given a disaster scenario including the data in the early hours after the disaster strike and a summarised list of humanitarian suppliers. The participant then needs to decide based on their own judgement and experiences which suppliers to choose for this particular disaster. However none of the participants actually came up with a list of decision (selected suppliers) or suggested any decision framework for disaster. This clarified further the lack of an established decision making framework for practitioners which was one of the reason for developing PREDIS. So the only output of this part is the lack of a reliable decision making framework related to this research. The questionnaire 1 is exhibited in Appendix A, Table A1. The data gathered in this questionnaire was then used to calculate the set of decisions by experts and non-experts through PREDIS.

#### 4.2.4. Post-Test

The result of the decisions based on the participants preferences was presented to the individuals. The expert participants were asked to fill in this questionnaire 2 about their opinion regarding the PREDIS model in comparison to the models they currently use. The non-expert participants currently do not have a model for supplier selection and therefore cannot compare it with the PREDIS model. However, they were asked about their opinion on the process of decision-making they experienced during the simulation game. The goal is to analyse the effect of expert’s background on their evaluation of the game. The components of the second questionnaire (feedback), is articulated in Table 4.

To summarise Table 4 helps to identify if the expert’s opinion has been affected by their sector, number of disasters in which they have participated, by comparing the results of their decisions with the other experts with different characteristics. The questionnaire also gives an idea about the existence, time effectiveness and confidence level of the existing decision frameworks they might currently use. The objective is to compare that further with the PREDIS model. To that end, the questionnaire also gathers data about the opinions of the decision maker towards the simplicity and time effectiveness of PREDIS and directly asks the experts if they will use/recommend the PREDIS model in a real situation and the reasoning behind their positive or negative answer. At the end, there is an opportunity for the decision makers to point out the areas of improvement for the PREDIS model. The questionnaire gathers data on four areas, the characteristics of the participants, existing framework, PREDIS framework, and the reasoning behind their comparison.

#### 4.2.5. Justification of the Questions within the Post-Test Design

The logic behind designing these questions are articulated as follows. *The characteristics of the existing framework* (existence, length, confidence level): The existence of a framework was asked because it was necessary to know if the experts already have a decision-making process in place to which they can compare the PREDIS. It was expected that the majority of the participants have them and these can be used further as a source of comparison and analysis of the PREDIS. The length of their current decision-making process was also asked because in the early hours after the disaster strike, the decisions regarding aid can be crucial. For example, the medical triage employs the “golden hour” rule. This is the period of time (first hour) in which the treatment of the patient in shock or with traumatic injuries is most critical [80]. In addition, the time for rescue can also be divided into the periods of less than 1 h, and 1–6 h [81,82]. In addition, time frame for providing the first action plan for providing critical resource needs is 12 h [83,84]. Therefore, the milestones for critical decisions to be made, for saving lives by medical triage (1 h), saving lives by rescue (6 h), and the action plan for critical resources needs (12 h), can be set. It was expected that the majority of the participants make their decisions under 6 h in order to be able to perform the initial rescue operations. However this would be one of the strength of PREDIS, where the decision makers have to decide within 6 h based on no information, PREDIS provides predicted values. The level of confidence was also asked about in order to see how much the decision makers require to rely on the PREDIS model as a source of their confidence due to the predictions it provides. It was expected that the majority of the participants would be confident enough to make decisions but not very confidant. 

*The characteristics of the PREDIS framework* (simplicity, real disaster, length): These questions were asked to specify if the PREDIS model could compete with the actual frameworks they are using at the moment. The few important points were, whether it is simple enough to be used under pressure, and by non-technical decision makers, also to make sure that the whole process does not supersede the critical time lines (1, 6, 12 h). In other words, make sure that the author’s assumption that the PREDIS can be used quickly by the decision makers is valid. It is expected that the participants find the PREDIS simple and quick to use and would use it in a real disaster, though some training might be required. The answer to these questions may signal the opportunity for the further expansion of PREDIS in the humanitarian sector. To that end the next level of questions are asked. 

*Possibility to expand PREDIS in practice* (recommendation, why yes, why no): At this point it is clearly asked if the participants would use PREDIS in a real disaster. The participants are prepared for this question in the previous question where they have thought about the strength and weaknesses of the model and have compared it with their existing framework. The answer to these questions was not clear at this point because it depends on the answers to the previous questions. However ideally the participants would use PREDIS and recommend it to others whilst clearly stating why. If this happens, then the author has a clear idea if the PREDIS model has met the requirements for which it was designed including being quick, using the data that are available at the time of the disaster, and taking into account the preferences of the decision maker. In addition, they might come up with some unforeseen reasons why they favour PREDIS. This would pave the way for developing PREDIS further into software and finding a market for its expansion. However, if the majority answer no, and they provide the reasoning behind their choice including that they believe PREDIS to be untrustworthy, complicated, unreal or any other reason, they would signal the necessity to revisit the model critically. 

To summarise, this questionnaire helps to identify if the expert’s opinion has been affected by their sector, number of disasters in which they have participated, by comparing the results of their decisions with the other experts with different characteristics. The questionnaire also gives an idea about the existence, time effectiveness and confidence level of the existing decision frameworks they might currently use. The objective is to compare that further with the PREDIS model. To that end, the questionnaire also gathers data about the opinions of the decision maker towards the simplicity and time effectiveness of PREDIS and directly asks the experts if they will use/recommend the PREDIS model in a real situation and the reasoning behind their positive or negative answer.

### 4.3. Debriefing and Data Analysis

The process of the game was then debriefed and data were analysed, the result of which will be explained in the result section. At the end, there is an opportunity for the decision makers to point out the areas of improvement for the PREDIS model.

## 5. Threats to the Validity

There are validity threats associated with this design [72], which affect the interpretation of the results as follows Table 5.

Table 5 shows that the internal validity can be affected by various factors. History can be a threat when some events occur between the pre-test and post-test which changes the course of the result. The effect of history is kept to a minimum by executing the process on one occasion. Therefore, the chance of events occurring which might lead to the change in measurements is reduced. Another threat is maturation, where the passage of time causes the responders to change (e.g., grow older, or get hungrier). This is also kept to a minimum by keeping the procedure short (45 to 90 min depending on the participants’ requirements) and by also offering breaks during the sessions. The testing effect occurs when the test is being taken is added to the scores of the previous tests. This is not applicable in this research because taking the second experiment does not depend on the score on the first experiment. 

The instrumentation effect happens when the changes in instruments or calibration of measuring happens. This is also kept to a minimum because the author runs all sessions herself and uses the same excel files, data case, presentations and computer systems. However, in some cases the sessions are held virtually on Skype, whereas in others the sessions are held in person. This is due to the geographical dispersion of the humanitarian workers involved, which made the in person sessions impossible. The statistical regression occurs when people are selected based on their high scores. This might be present in the research because the respondents are partly contacted based on their experience in the humanitarian field. However, measuring this effect is one of the secondary objectives of the study. So the presence of this threat will be measured later in the chapter. Any other discrepancies in the skills and capabilities of the respondents are non-intentional and therefore the selection biases are kept to the minimum. Selection biased happens when the groups are being selected based on different unequal measures. The loss of respondents during the sessions (experimental mortality threat) is unlikely during a 90-min session and therefore the mortality effect is kept to the minimum.

The external validity associated to this design includes multiple treatment and reactive/interactive effects effect of testing. Multiple exposures to treatments interfere with each other and the experience is not erasable from the mind of the participants. This is present in this research due to the design, which makes the participants exposed to the data of disaster in pre-test and post-test. Attempts have been made to even out both groups by switching the timing of the experiments and the Latin-square arrangement. This keeps the threat from contaminating the main effects of experiments [72]. However, the author is aware of this threat as a limitation of the study. The reactive/interactive effect of testing occurs when the participants are exposed to pre-test and this changes their sensitivity for the test variables and makes it unrepresentative of the untested group of participants. The reactive effects of experimental arrangements are also kept to the minimum by exposing the respondents to the treatments only in the experimental setting and not giving away data about the procedure of the experiment to the respondents before the sessions.

One important point, which is worthy to mention, is that the author initially aimed to assess the result of the simulation game using the Turing test [85]. Specifically the variation described as the “subject matter expert Turing test”, to see if the response of the machine (here the excel sheet embedded with the principals of the PREDIS model) is distinguishable from the expert’s. The process was designed in a way that the pre-test asks for experts’ specific decisions, then exposes them to the treatment (PREDIS model) and then uses the machine (computer) to generate the post-test result by incorporating the experts’ preferences. This test is also known as a “Feigenbaum test” [78]. However, the pilot study showed this test to be impossible to conduct, because experts would not give a clear set of decisions. For example, when asked “please rank and choose the suppliers you would use in the given disaster situation”, in the pre-test experts would say “I would use the suppliers with whom I have had good relationships in the past”, or “I would choose the suppliers based on the quality of previous experiences” or “I will call any local supplier in the area to see if they can provide the resources”. Therefore, comparing a list of chosen suppliers in the pre-test and post-test procedure was not possible. Consequently, the author ignored the use of Turing test and settled for the comparison of the result in the post-test between experts and non-experts. Therefore, the results of the pre-test in all cases were used just to show that at the moment an actual framework that provides clear comparable choices is non-existent. 

To summarise, the threats associated with the simulation game design in this research affect the internal and external validity as follows. Internal validity, which is present in this research, is regression biased, because the experts are selected based on their high level of experience in the disaster situation and this makes them unequal to the non-expert group. However, the difference in decision-making in these two groups is the subject of hypothesis (H2) and therefore it will be discussed in detail. The external validity is threatened by the reactive arrangement of experiments in addition to the multiple treatment interference. This is one of the most important limitations of the quasi-experiment design, which makes it less generalisable.

## 6. Results

In the pre-test phase of the simulation game, the participants were asked to rank a list of 20 suppliers. During the questionnaires the participants (experts) were also enquired about the frameworks they already have in place. Although a number of experts mentioned that they already have selection frameworks in place, none of them provided an actual ranking of the desired suppliers at this phase. For example, some experts mentioned HISS-CAM framework [86] is designed to ensure a balance combination of civil–military suppliers in a disaster response. This framework has been used for supplier selection in Afghanistan and Georgia amongst other countries, since 2008. It shows a flowchart were judgment calls need to be used to make sure the suppliers are aligned with the HISS principles. However, this does not provide numerical data about selecting the suppliers based on tasks. Another example experts mentioned is the American Red Cross cooperation with local churches in gathering the supplies from their warehouses (the expert was a former employee) which is done by calling the churches one by one to ensure the availability of the stock before sending out the trucks to bring their supplies. This is done on a first come first served basis and utilises the existing connections between the two entities (Red Cross and churches).

To that end, most of the existing frameworks for supplier selections mentioned in the process of simulation game, were understandably based on the elements of experience-based trust or resource-based choices. Meaning if the decision maker had previously worked with a supplier and trusted them, they were first in line to be called in, regardless of their current capabilities or the specific requirements of the disaster. In addition, if the decision maker knew, based on their experience, that some suppliers might be able to supply few resources, they were selected for participation. The result of the pre-test in two groups of expert and non-experts are incomparable to each other which is consistent with the findings in pilot study. The reason is that none of the participants could come up with an actual list of selected or ranked suppliers. This might be due to the lack of existing practical frameworks, which allow the calculation of numerical or ordinal values. This signals that most of the decisions in this area are done heuristically and as will be confirmed later in the result of the questionnaire 2 that these decisions are mostly experience-based rather than evidence-based. Therefore, only the results of the post-test for two groups of experts and non-experts will be compared which will be illustrated in the next part.

### 6.1. Result of the First Questionnaire

The first questionnaire collected data about the decision makers’ preferences in two groups of expert/nonexperts. In other words the results of the first questionnaire identify how the experts and non-experts prefer one supplier to another. The result of the questionnaire is analysed through a multi-attribute optimisation decision making using analytical hierarchy process (AHP) the full details of which can be found in the previous works of the authors in the EDIS framework of resource allocation and decision making. An example of the result of AHP weights calculated for experts and non-experts is illustrated in Table 6.

**Figure A1:**
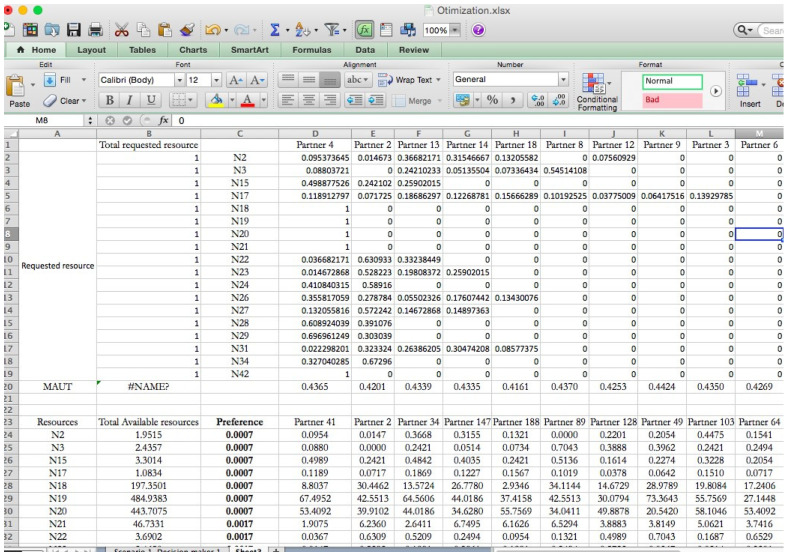
A snapshot of the optimised equation.

Table 6 shows that the experts’ preferences on average put more value on government (12%), and almost the same value on NGO (4.5%) and military (4.2%). On the other hand the non-experts put more value on military (8%) followed by government (7%), and volunteers (5.2%). Experts put more value on the small sized organisations (62%), whilst the non-experts gave the same value (3%) to small and very big organisations. Experts gave a high value for international expansion (65%), whilst non-experts had a low preference for international expansion (6%). Both groups had a high value for suppliers with more experience, however non-experts preferred experience (18%) to experts (10%). Both groups gave a higher value for the supplier with higher surge capacity, 8% for non-experts, and 6% for experts. However, for the lower surge capacities both values were around 2%. The experts gave the highest value for WASH (9.7%) shelter (7%), health (5%), and nutrition (35%) whilst non-experts gave the highest value for WASH (8.6%) health (5%) nutrition (36%) and shelter (2%). These preferences in combination with the resources available to the suppliers can be used to calculate the utility of each supplier using PREDIS [1,2,3,4,5,6] as articulated in Appendix B, Figure A1. This shows that in order to optimise the decision, the following resources need to be selected. For example 9.5% of the resource N2 should be obtained from supplier 4,whilst no units of N2 is obtained from supplier 8, 9, 3, 6.

#### Comparing the Result of the First Questionnaire

To compare the result between the two groups, a variation of outranking method associated to Borda [87] or Roy [88] is employed. The reason is that this is a classic multi-criteria decision making problem, where a set of alternatives is selected based on preferences expressed by decision maker. A common solution is to examine if partner (a) is as good as partner (b). The outranking techniques under this rule supported decision-making in voting [89], supplier selection [90] or project assessment [91] amongst others. Using Borda count the result of the first questionnaire for the group of experts is analysed as below. If a selection consists of a set (D) of Decision makers (here 22 decision makers for each group), each having a preference order for a set of (C) candidates (here 20 humanitarian supplier), the Borda rule here is calculated where a supplier receives n points each time they are selected as the most desirable, n-1 points when they are selected second to most desirable, and no points every time they are selected as the least desirable [90,91,92]. Here n is the number of candidates (here 20 suppliers) and 22 decision makers for each group of experts and non-experts. So using this technique, for experts, the Borda rule for supplier i can be calculated as follows. Experts have never (0 frequency) selected supplier 1 as their first choices (n = 20), so the Borda count is (0 × 20) = 0. Experts have never (0 frequency) selected supplier 1 as their second choices (n-1 = 19) so the Borda count is (0 × 19) = 0. Experts have twice (2 frequency) selected supplier 1 as their eighth choices (n-7 = 20-7) so the Borda count is (2 × 13) = 26. The total Borda count for supplier 1 is the sum of above individual Borda counts for supplier 1. An example of these results for experts is exhibited in Table 7

Table 7 shows that the total Borda count for supplier 1, 2, 3, 4 has been respectivel calculated as 144, 456, 326, and 500. This means that in this set, supplier 4 is the most desirable in the overall view of the experts. The final results of the Borda counts are calculated for all the 20 suppliers and are ranked in Table 8.

Table 8 shows that based on the Borda count, for the group of experts, supplier 4 who is a small military organisation with a high surge capacity, no international expansion, and low experience is the most desirable (with a 500 Borda count). Supplier 9, who is a medium sized government organisation with no expansion, and a high degree of experience and surge capacity is the least desirable (with a 105 Borda count). The same process has been repeated for the non-expert group and the results are exhibited in Table 9.

Table 9 shows that the non-experts preferred supplier 5 and 12 equally (333 Borda count) mostly because they are both small governmental organisations, with international expansion. It seems that the non-experts care less about the surge capacity and experience. Their least favourite are suppliers 20 and 17 with (a 135 and 136 Borda count), who are very big organisations with international expansion, and low surge capacity and experience. As far as the comparison of first and last choices of the experts and non-experts reveals, there is no evidence that by using the PREDIS model these two groups make the same choices. However, the NRMSE has been used to calculate a more precise percentage of error between the choices of the two groups. The NRMSE for difference between the two is calculated as 29% (Error between non-experts and experts) and 14% (Error between experts and non-experts). This means that at least 14% and at most 29% of the times, the nonexperts’ choices are different from the experts. This also means although the first and last choice of the majority of decision makers in the two groups are not the same, between 71% and 86% of the times experts and non-experts decide similarly using the PREDIS framework.

The significance of this result is that the non-expert does so with no prior training or data other than the data that are freely available on the Internet through the UN related and World Bank related websites (including HDI, DRI, population, population density, and disaster type). Therefore, it is possible to conclude that although the result shows that the experts and non-experts may have various preferences, the model enables the non-experts to choose suppliers similarly to experts, if necessary.

### 6.2. Comparing the Result of the Second Questionnaire

The second questionnaire was only given to the experts because as was mentioned before they needed to evaluate the PREDIS model with the existing models they had in place. This situation does not exist for non-expert so giving the second questionnaire to them would be meaningless. An example of the accumulated data from questionnaire 2 is exhibited in Appendix C (Table A2) for two exemplary experts. This shows that for example expert 1 who is over 50 years of age and has experience in working with NGO s and the military in one International disaster and mostly national US disasters, does not have a formal framework for decision-making. Furthermore, s/he is not extremely detailed about whether s/he is confident about this informal framework enough to make decision, however s/he believes that the big suppliers are biased towards their decisions and because they do not want to lose, they overestimate their decision capabilities. For this reason, s/he prefers the small suppliers to the big ones. S/he also believes that although the PREDIS model is complicated, it is time effective, and the time required for performing it, will considerably decrease with practice. S/he is able to use PREDIS if receiving training before the disaster strikes, however s/he believes that most of the decision makers will say they will not have time to use PREDIS in a real disaster situation.

The second expert who is younger (between 35 and 50) has experience of working with NGOs in more than five international disasters. Because s/he is operational, does not have a framework for decision-making per se, but s/he uses some guidelines, specific around capabilities/radio supplier with locals, which takes less than 12 h to perform. S/he believes that PREDIS is simple but time consuming and knowing your organisation is more important. This person is not interested in the supplier selection part of the PREDIS, but very interested in the tangible information, which the predictive part of the PREDIS can provide about the amount of needs. In fact, this conversation with this expert led to a suggestion for cooperation with the author to develop PREDIS into a real time software program in the future. S/he also believes predicting a range helps a lot as long as the range, is between 100 and 150,000. This answer, which is confirmed by several other experts, is very important because it further assured the author that giving the range for the predictions, is not a limitation of PREDIS, but can be considered a strength from the experts’ point of view. The result of the second questionnaire is presented in Table 10.

The result of the Table 10 can be interpreted as follows.

*The characteristics of the expert group*—Although experts had experience in variety of humanitarian organisations, had experience of national disasters in their country, not exclusive to the experiences of working in international disasters. These characteristics give a wide range of expertise and perspectives to the simulation game.

*Characteristics of existing frameworks*—The majority of the experts (68%) had frameworks in place for choosing suppliers. However, they mostly rely on heuristics accounts of trust, previous experiences, self-declared resources, and capabilities and the respected guidelines are mostly generic. None of which contained numerical and measurable guidelines. For example, when choosing military suppliers they used guidelines such as the guideline European interagency security forum presents (eisf.eu, 2014). Therefore, the author concludes that in practice a specific numerical and measurable guideline, which can clearly compare various suppliers, is missing. Further investigation regarding the existing frameworks is required which can be the subject of another study.

*Characteristics of PREDIS model*—Majority of the experts (73%) thought that the PREDIS model was simple to use, and therefore will use PREDIS in a real situation (86%) if they have training beforehand. Although some will not use, all the experts (100%) believed that given prior training, a decision maker is able to use PREDIS model in the disaster situation without the aid of the facilitator, and make decisions within an hour. It is noteworthy to mention these reasons are not exclusive and experts could choose more than one reason. *Possibility to expand PREDIS in practice*—the experts provided various suggestion for the expansion of PREDIS. Including the importance of the primary supplies such as water and sanitation, and menstrual hygiene over the secondary products like shampoo and toothpaste. So further research needs to be done to confirm the level of necessity of these product through the relationship with host communities in order to get needs assessment. Introducing some elements of risk to the model where the severity of the disaster, could be the subject of an extensive research as well as adding more weights to the essential supplies. The exact weight for this calculation however could be the subject of further studies. Also the model on different strategic levels of strategic or a pyramid structure to define the three essential elements of analysis were suggested. Other suggestions include considering the capabilities of the individuals, mobilisation time, and differentiate between local suppliers and small suppliers when it comes to setting the preferences. Because the local suppliers have quicker access, to the population in need. Another suggestion was to use the model in actual cases to measure its usefulness. Some stated that the model is unique compared to the existing incident management software. One expert said that the model is not useful at the time of the disaster but good for scenario planning before a disaster. All the experts confirmed that having a range of predictions could help them plan better than if they have one solid number as prediction.

## 7. Conclusions and Limitation of the Research

The aim of this research was to test the suitability of the PREDIS model further for decision-making in the disaster situation. It was initially expected that the majority of the participants have their own decision model. Using a simulation game in a frame of a quasi-experiment, two series of expert and non-expert replayed a hypothetical scenario of disaster response resource allocation. The decisions made by the two groups were registered and compared to examine two hypothesis.

### 7.1. Conclusions

**Hypothesis** **1.** *Inquired if ‘The PREDIS model assists the decision makers in making the same decisions faster’*.

The simulation game confirms that the experts (100%) agreed on given prior training, a decision maker is able to use PREDIS model in the disaster situation without the aid of a facilitator and make decisions within an hour.

**Hypothesis** **2.** *inquired that ‘The PREDIS model assists the non-experts in making decisions as well as experts’*.

Although the first and last choices of the experts and non-experts are not the same, in 71–86% of the times, experts and non-experts decide similarly using the PREDIS framework. The significance of this result is that the non-expert does so with no prior training or data other than the data, which are freely, available on the Internet through the UN related and World Bank related websites (REF to PRED). Therefore, it is possible to conclude that although the experts and non-experts may have various preferences, the model enables the non-experts to choose suppliers similarly to experts, if necessary.

The overall results were analysed in two parts. The numerical results of the decisions show that the PREDIS model has two major capabilities. It enables the experts and non-experts to predict the disaster results immediately and using the widely available data. It also enables the non-experts to decide almost the same as the experts; either in predicting the human impact of the disaster and estimating the needs or in selecting suitable suppliers. It is also the only framework of its type, which takes specific numerical values as input, and provides specific numerical values and clear decisions as outputs such as which suppliers to supply how many units of requirements. The result also shows that even the experts who have frameworks in place (two of them were described earlier) mostly rely on heuristics accounts of trust, previous experiences, self-declared resources, and capabilities. Therefore, the conclusion can be drawn that in practice a specific numerical and measurable guideline, which can clearly compare various suppliers, is missing. Second it was initially expected that the majority of the participants make their decisions under 6 h (golden hour) in order to be able to perform the initial rescue operations. The result shows that without the PREDIS model, 23% of the experts take less than one-hour to make decisions, 45% take between 1–6 h to make decisions, and 32% take more than 12 h to make a decision. However using the PREDIS model all the participants could make their decision in less than an hour. This further confirms that the PREDIS model assist decision makers to make faster decisions.

There are secondary results that can be drawn also shows that the experts’ preferences on average put more value on government (12%), and almost the same value on NGO (4.5%) and military (4.2%). On the other hand the non-experts put more value on military (8%) followed by government (7%), and volunteers (5.2%). Experts put more value on the small sized organisations (62%), whilst the non-experts gave the same value (3%) to small and very big organisations. Experts gave a high value for international expansion (65%), whilst non-experts had a low preference for international expansion (6%). Both groups had a high value for suppliers with more experience, however non-experts preferred experience (18%) to experts (10%). Both groups gave a higher value for the supplier with higher surge capacity, 8% for non-experts, and 6% for experts. However, for the lower surge capacities both values were around 2%. The experts gave the highest value for WASH (9.7%) shelter (7%), health (5%), and nutrition (35%) whilst non-experts gave the highest value for WASH (8.6%) health (5%) nutrition (36%) and shelter (2%).

Another part of analysis is associated with the question answered exclusively by experts. The conclusion drawn from this questionnaire is that although the experts already have their own heuristic frameworks, they are positive towards using the PREDIS model in real situations if they have prior training. This is because of the speed of PREDIS model, its relative simplicity, its use of available data, its predictive ability, and its clear decision outputs.

### 7.2. Limitation of the Research

There are some limitations associated with the model. First, it is purely theoretical at the moment and has yet to be tested in a real disaster situation. Also the initial plan was to provide the MIRA report in the pre-test to get the decision maker to decide based on the information available 72 h after the disaster strike. Then in the post-test give them the PREDIS framework which needs no information about the disaster in real-time and compare the result and see to what extent the selected suppliers are similar and therefore draw the conclusion that whether PREDIS makes decision makers to make the same decisions faster. However this could not happen because in the pre-test no participant selected actual comparable suppliers. The decision process in this phase was vague and was rarely based on the non-numerical guidelines. To that end, comparing the set of suppliers in pre-test and post-test rendered it impossible. However the author still argues that the fact that most of the participants said they could use PREDIS within one hour to decide whilst their current decision-making process takes five hours on average, is an indication that the PREDIS model helps the decision maker to decide faster and therefore bridges the gap between the time the decision is required and the time that the data becomes available.

Second, the research shows that comparing to the existing decision models in humanitarian sector the PREDIS could prevails the existing guideline which either are vaguely based on flow charts of qualitative judgment calls from the decision maker’s part (IESF) or are based predominantly on highly specialised data (HAZUS). In a sense, the model gives numerical choices of suppliers whilst it is using simple available data usable for people with the least technical background.

Third, in addition, based on the expert’s opinion and the initial research, the PREDIS compared to the existing decision models in the commercial sector such as incident management and business continuity software (CIRmagazine.com, 2014) has a better predictive capability without accessing to the real-data feed, which is difficult or impossible to obtain in a disaster situation, especially in less developed countries with a lower level of communicative infrastructures.

## 8. Contribution and Future Research Direction

The contributions of the research was the evaluation of DSS framework called PREDIS through a simulation game, which was conducted as a quasi-experiment using expert and non-expert participants. The results show that the PREDIS model’s significance is threefold. First, it is the first decision framework of its type that enables the decision maker to predict and estimate the needs and select the suppliers using the data that are readily available for each country at the time of the disaster. It also enables non-experts to make decisions almost as well as experts in a disaster situation. Moreover, it enables experts and non-experts to make decisions within one hour after the disaster strike using the limited data available before and immediately after the disaster strike.

The contribution to theory is a unique insight into the growing body of research that examines the proliferation problem in a disaster response network. The research also is one of the pioneers in using a simulation game design for incorporating the human agents’ opinions into the model. In that aspect, it integrates the hard and soft decision techniques within the concept of Systems thinking theory. Although the use of a combination of Resource-dependency theory and Decision theory is common practice in the literature, the combination of the above theories, in order to improve the collaborative success in short-term disaster operations is rare despite its extensive use in the medical and psychological field of decision-making. Although by using simulation game design the research enters the area of operational behaviour to some extent, due to the recent development of this discipline, further research is required to confirm the conformity of this model within this discipline. The complementarities of the above capabilities of the research may reinforce earlier studies and provide a valuable contribution to the understanding of the complex mechanisms of relationships between the determinants of the disaster impact, the way the expert and nonexpert decision makers think and decide, and the effect of re-structuring the disaster response network.

It also provides a number of methodological implications. This research uses two phases for validation of the PREDIS model. First, it uses the hypothetical scenarios to show the mechanism of the model and it identifies whether the model works in its own right, then provides a simulation game design to simulate the decision-making under uncertainty in the disaster situation by taking into account the opinion of human agents. This is as well as differentiating between two groups of human agents: Experts and non-experts. By putting forward the results of the resulting decisions from both groups, the research enables the researcher to identify how the decision-making could be different using different agents from different backgrounds. It also uses mathematical optimisation in addition to the opinion of human agents, which is in accordance with the background of the research, which integrates the heuristic and mathematical approaches of decision-making.

Overall, the research fills the gap in the fledgling field of disaster management, especially by enriching the predictive power of the decision maker. This give rise to the practical contribution enables the experts and non-experts to customise their decision-making process by entering their personal preferences into the process regardless of their experience, knowledge, first, for example, the model is based on the resources-based optimisation, it takes into account the decision makers’ preference and characteristics in various other criteria such as experience, type, and size of the organisation, its surge capacity, and international expansion. Further research is required to identify the actual non-resource based determinants of supplier selection in collaborative networks with the focus on disaster response. Also the model could be combined with business continuity software in order to give rise to the planning and actions after decision-making. The investigation and comparison through existing software suitable for this purpose could be the subject for an extensive research. Also assessing the quality of the decisions by (non)experts well as the motivation behind these decisions could be the subject of future research.

## Figures and Tables

**Table 1:** An example of the secondary panel data adapted from PRED utilised in Input 1a.

Dis. No.	End	Country	Name	Country’s HDI	Country’s DRI	Impact Time	Population	Population Density	Killed	Total Affected
2013-0386	17/08/2013	Nigeria	General Flood	0.47	8.28	125	168,833,776	180.28	19	81,506
2013-384	13/08/2013	Gambia The	General Flood	0.44	11.84	1	1,791,225	171.44	2	3300
213-0378	21/02/2013	Philippines	Tropical Cyclone	0.65	27.98	1	96,706,764	318.79	6	262,884

**Table 2:** An example of the secondary cluster-based data adapted from EDIS utilised in Input 1c.

	WASH Cluster Needs		Nutrition Cluster Needs		Shelter Cluster Needs		Health Cluster Needs	
	200 g Soap	Water for Patients	Canned Fish	Pasta	Rope	Shovel	Doctors	Nurses
Partner 1	28	6	14	42	34	33	69	2
Partner 2	46	63	43	76	87	55	62	62
Partner 3	46	55	19	20	27	95	41	90

**Table 3:** The design of the game.

Category	Sub-Category	In this Paper
Participant characteristics prior to game play	Conceptual pre-requisite	conceptual decision makers in disaster decision
Output through a questionnaire 1	Skill pre-requisite	two groups of experts and non-experts in disaster management
Game administration factors	Group/individual decision-making	Individual
Input through Game protocol	Intermittent and structured discussion	discussion after simulation game
	Pacing	time for each session is between 45 to 90 min
	Group size	two groups of 22 participants
Game Structural factors	Written decision-making records	excel files showing the process
	Predicting accurate feedback	Feedback compared in both groups answering hypothesis H1
	Similarity of problem and data presentation	data about the decisions taken by participants are presented in the same units (of aid required) and suppliers (selected for the response) as was presented in the problem provided at the beginning of the game.
	Decision-making procedure specificity	The game protocol including the PREDIS is followed by both groups of participants.The output is two sets of decisions. The result of the 2 questionnaires in post and pre-test phase will be analysed.

**Table 4:** Components of second questionnaire.

Goal	Question Category	Criteria	Sub Criteria	Expected Response
Analysing the effect of expert background on their evaluation of the PREDIS model	Participant’s characteristics	Age	<35	It was expected from the respondents who have expressed their initial interest in participation, that the respondents are experienced, meaning they are older than 35, with experience in various sectors and in both international and national disasters.
			35 to 50	
			>50	
		Sector	Public humanitarian	
			NGO	
			Government	
			Military	
			Other	
		Number of Disaster	1 International	
			1 to 5 international	
			More than 5 international	
			Just national	
	Existing framework characteristics	Existing Framework	Yes	It was expected that most of the participants have frameworks in place and it takes them less than 12 h to decide and have enough confidence in their framework to decide.
			No	
		How long to use the existing framework	<1 h	
			1 to 6 h	
			<12 h	
		Confidence level in the existing framework	Very	
			Enough to decide	
			Better than nothing	
			Impossible to be confident	
	PREDIS framework characteristics	Simplicity of PREDIS	Simple/time effective	It is expected that the participants find the PREDIS simple and quick to use and use it in real disaster and recommend it to others.
			Simple/Time consuming	
			Complicated/ti me effective	
			Complicated/ti me consuming	
		Use of PREDIS in real disaster	Yes, instruction	
			No due to time	
			No, use my own	
			No, other reason	
		How long it takes to implement PREDIS in real situation	<1 h	
			<6 h	
			<12 h	
	Possibility to expand PREDIS in practice	Future use/recommendation of PREDIS	Yes	The answer to these questions is not clear at this point, because it depends on the comparison with the previous stage.
			Yes, recommend	
			No	
			No, recommend	
		Why yes	Better than nothing	
			Quick	
			Available data	
			Preference	
			Others	
		Why no	Vague	
			Untrustworthy	
			Unrealistic	
			Complicated	
			None	
			Areas of improvement	

**Table 5:** Threats to the validity of this simulation game design.

Design Validity	Threats to the Design Validity	Addressed
Internal validity	History	Unlikely
	Maturation	Reduced
	Testing	Not applicable
	Instrumentation	Reduced
	Regression	Yes
	Selection	Reduced
	Mortality	Unlikely
External validity	Interaction of testing and experiment	Reduced
	Interaction of selection and experiment	Reduced
	Reactive arrangement	Yes
	Multiple treatment interference	Yes

**Table 6:** Comparing AHP preferences/experts and non-experts.

Level 1	Level 2	Non Expert	Expert
International Expansion	Yes	0.06	0.653
	No	0.022	0.005
Experience	Low	0.026	0.042
	Medium	0.039	0.045
	High	0.066	0.06
	Expert	0.177	0.099
Surge capacity	Low	0.025	0.027
	Medium	0.029	0.022
	High	0.025	0.029
	Very high	0.074	0.06
WASH	Transportation container (10–20 lit)	0.086	0.097
	Storage container (10–20 lit)	0.086	0.097
	250 g bathing soap	0.086	0.097
	200 g laundry soap	0.086	0.097
	Acceptable material for menstrual hygiene	0.086	0.097
	Blanket	0.086	0.097
	75 mL/100 g toothpaste	0.086	0.097
	One toothbrush	0.086	0.097
	250 mL shampoo	0.086	0.097
	250 mL lotion for infants and children up to 2 years of age	0.086	0.097
	One disposable razor	0.086	0.097
	Underwear for women and girls of menstrual age	0.086	0.097
	One hairbrush and/or comb	0.086	0.097
	Nail clippers	0.086	0.097
	Total basic water needs	0.086	0.097
	Water for patients	0.086	0.097
	Water tap	0.086	0.097
	Hand Pump	0.086	0.097
	Open well	0.086	0.097
	Toilets	0.086	0.097
	Trench latrines,	0.086	0.096
Nutrition	SALT, iodised edible	0.036	0.035
	SUGAR, white	0.036	0.035
	YEAST, dried, package 11 gr	0.036	0.035
	FISH, canned, sardines, veg oil, 150 g	0.036	0.035
	PASTA, durum wheat meal	0.036	0.035
	RICE, white, long grain, irri6/2	0.036	0.035
	OIL, rapeseed	0.036	0.035
	BEANS, white, small	0.036	0.035
Shelter Cluster	Tarpaulin (4 m × 6 m)	0.023	0.067
	Rope (30 m)	0.023	0.067
	Saw	0.023	0.067
	Roding, small and largo Nail (1/2 kg each)	0.023	0.067
	Shovel	0.023	0.067
	Hoe	0.023	0.067
	Machete	0.023	0.067
	Shear	0.023	0.067
	Wire (1.5 mm diameter) meter	0.023	0.067
	Claw hammer	0.023	0.067
	Woven Sack	0.023	0.067
Level 1	Level 2	Non Expert	Expert
Health Cluster	Doctors	0.054	0.047
	Nurses	0.054	0.047
	Other specialties	0.054	0.047

**Table 7:** Example of the Borda count for the group of experts.

Choice Rank	N	Supplier 1		Supplier 2		Supplier 3		Supplier 4	
		Frequency	Borda	Frequency	Borda	Frequency	Borda	Frequency	Borda
1st	n	0	0	2	40	4	80	15	300
2nd	n-1	0	0	3	57	4	76	5	95
3rd	n-2	0	0	2	36	3	54	1	18
4th	n-3	0	0	9	153	2	34	0	0
5th	n-4	0	0	1	16	4	64	2	32
6th	n-5	0	0	2	30	0	0	0	0
7th	n-6	0	0	0	0	0	0	0	0
8th	n-7	2	26	0	0	1	13	0	0
9th	n-8	0	0	0	0	0	0	0	0
10th	n-9	0	0	1	11	0	0	0	0
11th	n-10	0	0	0	0	0	0	1	10
12th	n-11	3	27	0	0	0	0	1	9
13th	n-12	1	8	0	0	0	0	1	8
14th	n-13	0	0	1	7	0	0	0	0
15th	n-14	3	18	1	6	0	0	0	0
16th	n-15	13	65	0	0	0	0	0	0
17th	n-16	0	0	0	0	0	0	1	4
18th	n-17	0	0	0	0	0	0	0	0
19th	n-18	0	0	0	0	1	2	7	14
20th	n-19	0	0	0	0	3	3	10	10
Total Borda count			144		356		326		500

**Table 8:** Expert borda count ranking.

Supplier	Borda Count	Type	Size	Expansion	Experience	Surge Capacity
Supplier 4	500	Military	Small	No	Low	Very high
Supplier 5	427	Government	Small	Yes	Low	Low
Supplier	Borda count	Type	Size	Expansion	Experience	Surge capacity
Supplier 2	356	Military	Small	Yes	Low	Medium
Supplier 16	344	Government	Medium	Yes	Low	Very high
Supplier 3	326	Volunteer	Medium	Yes	High	High
Supplier 7	294	Volunteer	Small	Yes	High	Medium
Supplier 17	292	International	Very big	Yes	Very high	Medium
Supplier 18	283	Government	Small	Yes	Low	Very high
Supplier 8	271	Volunteer	Very big	Yes	Low	High
Supplier 12	252	Government	Small	Yes	High	High
Supplier 15	250	International	Big	Yes	Low	High
Supplier 20	231	Government	Very big	Yes	Low	Low
Supplier 19	203	Volunteer	Small	Yes	Very high	Medium
Supplier 13	187	Volunteer	Small	Yes	Medium	Low
Supplier 10	175	Volunteer	Small	Yes	High	Very high
Supplier 14	151	Government	Small	No	Low	Low
Supplier 1	144	Government	Big	Yes	Very high	Low
Supplier 11	142	Government	Very big	Yes	Low	Medium
Supplier 6	114	NGO	Small	No	Very high	Low
Supplier 9	105	Government	Medium	No	High	Very high

**Table 9:** Non-expert Borda count ranking.

Supplier	Borda Count	Type	Size	Expansion	Experience	Surge Capacity
Supplier 5	333	Government	Small	Yes	Low	Low
Supplier 12	333	Government	Small	Yes	High	High
Supplier 7	326	Volunteer	Small	Yes	High	Medium
Supplier 2	318	Military	Small	Yes	Low	Medium
Supplier 3	280	Volunteer	Medium	Yes	High	High
Supplier 10	259	Volunteer	Small	Yes	High	Very high
Supplier 18	259	Government	Small	Yes	Low	Very high
Supplier 16	258	Government	Medium	Yes	Low	Very high
Supplier 19	238	Volunteer	Small	Yes	Very high	Medium
Supplier 4	233	Military	Small	No	Low	Very high
Supplier 14	224	Government	Small	No	Low	Low
Supplier 6	205	NGO	Small	No	Very high	Low
Supplier 9	196	Government	Medium	No	High	Very high
Supplier 13	195	Volunteer	Small	Yes	Medium	Low
Supplier 15	186	International	Big	Yes	Low	High
Supplier 8	177	Volunteer	Very big	Yes	Low	High
Supplier 1	174	Government	Big	Yes	Very high	Low
Supplier 11	155	Government	Very big	Yes	Low	Medium
Supplier 17	136	International	Very big	Yes	Very high	Medium
Supplier 20	135	Government	Very big	Yes	Low	Low

**Table 10:** The accumulated result of the second questionnaire.

Participants Information	Options	Number of Responses	Percentage
The responder’s age	a. Under 35	1	
	b. Between 35 to 50	12	
	c. Over 50	9	
The respondent’s sector experience:	a. Public humanitarian organisations	1	
	b. NGO	7	
	c. Non-military part of a government	6	
	d. Military	2	
	e. Others (please explain)	5	
The respondent’s experience in previous disasters:	a. One international disaster	2	
	b. Between one and five international disasters	2	
	c. More than five international disasters	9	
	d. Just national disasters	11	
1. Have you had a framework for supplier selection in previous disaster situations?	a. Yes	15	68.18%
	b. No	7	31.82%
2. If yes, how long does it take to perform this framework in real situation?	a. Less than one hour	5	22.73%
	b. Less than five hours/Not extremely detailed	10	45.45%
	c. Less than 12 h	0	0.00%
	d. More than 12 h	7	31.82%
3. How confident are you about the result of the decision from your existing framework?	a. Very confident	5	22.73%
	b. Confident enough to make a decision	17	77.27%
	c. Not so confident/better than no framework.	0	0.00%
	d. It is against the nature of a disaster to be confident about any decision.	0	0.00%
4. How simple were to make you familiarise with the new model?	a. Relatively simple and time effective	13	59.09%
	b. Relatively simple but time consuming	3	13.64%
	c. Complicated but time effective	6	27.27%
	d. Complicated and time consuming	0	0.00%
5. Will you be able to perform this model at the real disaster situation?	a. Yes, if I have the detailed instruction	19	86.36%
	b. No, because I will not have time at the disaster situation.	1	4.55%
	c. No, because I will use my own framework.	0	0.00%
	d. No, for other reasons (please explain).	2	9.09%
6. How long does it take to perform the new model without the help of the facilitator?	a. Less than one hour	22	100.00%
	b. Less than five hours	0	0.00%
	c. Less than 12 h	0	0.00%
	d. More than 12 h	0	0.00%
7. Do you find this model helpful?	a. Yes	2	9.09%
	b. Yes, and I would recommend to colleagues.	15	68.18%
	c. No, I would not recommend to colleagues.	0	0.00%
	d. No but I would recommend to colleagues.	5	22.73%
8. If yes what are the reasons? (You can choose one or all the answers).	a. There is finally one guideline I can use.	0	0.00%
	b. It is quick to perform.	22	100.00%
	c. It uses available data.	5	22.73%
	d. It accommodates my preferences.	3	13.64%
	e. None of the above (Please explain)	3	13.64%
9. If no (if you will not use it), what are the reasons?	a. It is vague.	0	0.00%
	b. I can’t trust the procedure.	0	0.00%
	c. It is not realistic (not close to the real situation of disaster).	0	0.00%
	d. It is complicated to use.	0	0.00%
	e. None of the above (please explain)	3	13.64%
10. Would you lend us some time and identify the areas of improvement in the model?	Various comments	0	0.00%

## Data Availability

The dataset would be available upon request.

## Appendix A

**Table A1:** The questionnaire 1.

1. In Respect to the Type of the Suppliers:																		
	How Much More Important								Equal	How Much Less Important								
Government	9	8	7	6	5	4	3	2	1	2	3	4	5	6	7	8	9	NGO
Government	9	8	7	6	5	4	3	2	1	2	3	4	5	6	7	8	9	Military
Government	9	8	7	6	5	4	3	2	1	2	3	4	5	6	7	8	9	International
Government	9	8	7	6	5	4	3	2	1	2	3	4	5	6	7	8	9	Volunteers
NGO	9	8	7	6	5	4	3	2	1	2	3	4	5	6	7	8	9	Military
NGO	9	8	7	6	5	4	3	2	1	2	3	4	5	6	7	8	9	International
NGO	9	8	7	6	5	4	3	2	1	2	3	4	5	6	7	8	9	Volunteers
Military	9	8	7	6	5	4	3	2	1	2	3	4	5	6	7	8	9	International
Military	9	8	7	6	5	4	3	2	1	2	3	4	5	6	7	8	9	Volunteers
Volunteer	9	8	7	6	5	4	3	2	1	2	3	4	5	6	7	8	9	International
2. In respect to the size of the suppliers being (based on ANLAP, 2012)																		
Small	9	8	7	6	5	4	3	2	1	2	3	4	5	6	7	8	9	Medium
Small	9	8	7	6	5	4	3	2	1	2	3	4	5	6	7	8	9	Big
Small	9	8	7	6	5	4	3	2	1	2	3	4	5	6	7	8	9	Very big
Medium	9	8	7	6	5	4	3	2	1	2	3	4	5	6	7	8	9	Big
Medium	9	8	7	6	5	4	3	2	1	2	3	4	5	6	7	8	9	Very big
Big	9	8	7	6	5	4	3	2	1	2	3	4	5	6	7	8	9	Very big
3. In respect to the experience of the suppliers being																		
Low	9	8	7	6	5	4	3	2	1	2	3	4	5	6	7	8	9	Medium
Low	9	8	7	6	5	4	3	2	1	2	3	4	5	6	7	8	9	High
Low	9	8	7	6	5	4	3	2	1	2	3	4	5	6	7	8	9	Expert
Medium	9	8	7	6	5	4	3	2	1	2	3	4	5	6	7	8	9	High
Medium	9	8	7	6	5	4	3	2	1	2	3	4	5	6	7	8	9	Expert
High	9	8	7	6	5	4	3	2	1	2	3	4	5	6	7	8	9	Expert
4. In respect to the suppliers’ surge capacity (the ability to rapidly expand beyond normal capacity to meet the increased demand)																		
None	9	8	7	6	5	4	3	2	1	2	3	4	5	6	7	8	9	Low
None	9	8	7	6	5	4	3	2	1	2	3	4	5	6	7	8	9	Medium
None	9	8	7	6	5	4	3	2	1	2	3	4	5	6	7	8	9	High
Low	9	8	7	6	5	4	3	2	1	2	3	4	5	6	7	8	9	Medium
Low	9	8	7	6	5	4	3	2	1	2	3	4	5	6	7	8	9	High
Medium	9	8	7	6	5	4	3	2	1	2	3	4	5	6	7	8	9	High
5. In respect to the suppliers’ international expansion																		
Yes	9	8	7	6	5	4	3	2	1	2	3	4	5	6	7	8	9	No
6. In respect to the humanitarian clusters of the needs, do you prefer the suppliers to provide any particular need to the other clusters?																		
WASH cluster	9	8	7	6	5	4	3	2	1	2	3	4	5	6	7	8	9	Nutrition cluster
WASH cluster	9	8	7	6	5	4	3	2	1	2	3	4	5	6	7	8	9	Shelter cluster
WASH cluster	9	8	7	6	5	4	3	2	1	2	3	4	5	6	7	8	9	Health cluster
Nutrition cluster	9	8	7	6	5	4	3	2	1	2	3	4	5	6	7	8	9	Shelter cluster
Nutrition cluster	9	8	7	6	5	4	3	2	1	2	3	4	5	6	7	8	9	Health cluster
Shelter cluster	9	8	7	6	5	4	3	2	1	2	3	4	5	6	7	8	9	Health cluster
7. In respect to the above decision criteria, which one is more important to you?																		
Type	9	8	7	6	5	4	3	2	1	2	3	4	5	6	7	8	9	Size
Type	9	8	7	6	5	4	3	2	1	2	3	4	5	6	7	8	9	Experience
Type	9	8	7	6	5	4	3	2	1	2	3	4	5	6	7	8	9	Surge capacity
Type	9	8	7	6	5	4	3	2	1	2	3	4	5	6	7	8	9	International Expansion
Type	9	8	7	6	5	4	3	2	1	2	3	4	5	6	7	8	9	Cluster
Size	9	8	7	6	5	4	3	2	1	2	3	4	5	6	7	8	9	Experience
Size	9	8	7	6	5	4	3	2	1	2	3	4	5	6	7	8	9	Surge capacity
Size	9	8	7	6	5	4	3	2	1	2	3	4	5	6	7	8	9	International Expansion
Size	9	8	7	6	5	4	3	2	1	2	3	4	5	6	7	8	9	Cluster
Experience	9	8	7	6	5	4	3	2	1	2	3	4	5	6	7	8	9	Surge capacity
Experience	9	8	7	6	5	4	3	2	1	2	3	4	5	6	7	8	9	International Expansion
Experience	9	8	7	6	5	4	3	2	1	2	3	4	5	6	7	8	9	Cluster
Surge capacity	9	8	7	6	5	4	3	2	1	2	3	4	5	6	7	8	9	International Expansion
Surge capacity	9	8	7	6	5	4	3	2	1	2	3	4	5	6	7	8	9	Cluster
International Expansion	9	8	7	6	5	4	3	2	1	2	3	4	5	6	7	8	9	Cluster

## Appendix C

**Table A2:** Example of accumulated data in second questionnaire.

Participants Information	Options	Expert 1		Expert 2
The responder’s age	a. Under 35			
	b. Between 35 to 50			1
	c. Over 50		1	
The respondent’s sector of experience:	a. Public humanitarians			
	b. NGO		1	1
	c. Non-military part of a government			
	d. Military		1	
The respondent’s experience in previous disasters:	a. 1 international disaster		1	
	b. Between one and five international disasters			
	c. More than five international disasters			1
	d. Just national disasters/US	US		
1. Have you had a framework for supplier selection in previous disaster situations?	a. Yes			
	b. No	Not a formal one		B No (I am operation, some guidelines, specific around capabilities/radio supplier with locals).
2. If yes, how long does it take to perform this framework in real situation?	a. Less than one hour			
	b. Less than five hours/Not extremely detailed	Not extremely detailed		
	c. Less than 12 h			
	d. More than 12 h		1	
3. How confident are you about the result of the decision from your existing framework?	a. Very confident			
	b. Confident enough to make a decision	Biased, they have a commitment biased, stick to the commitment, because they don’t want to lose. Overestimate their decision capabilities.	1	
	c. Not so confident but is better than no framework.			
	d. There is against the nature of the disaster to be confident about any decision at the time of the disaster.			
4. How simple were to make you familiarise with the new model?	a. Relatively simple and time effective			
	b. Relatively simple but time consuming		Understanding of your own organisation is more important, content critical; military shows how that is critical.	
	c. Complicated but time effective	Completely will decrease by practice.		
	d. Complicated and time consuming			
5. Will you be able to perform this model at the real disaster situation?	a. Yes, if I have the detailed instruction	Prior to disaster		
	b. No because I will not have time in the disaster situation.	Most decision makers say		
	c. No because I will use my own framework.			
	d. No, for other reasons (please explain).		Complicated, tangible information, not interested in supplier selection, predicting damage and e1trapolating the amount of needs. Range helps, 100–150,000 helps.	
